# *USH2A*-retinopathy: From genetics to therapeutics

**DOI:** 10.1016/j.exer.2020.108330

**Published:** 2020-12

**Authors:** Lyes Toualbi, Maria Toms, Mariya Moosajee

**Affiliations:** a Development, Ageing and Disease, UCL Institute of Ophthalmology, London, EC1V 9EL, UK; b Ocular Genomics and Therapeutics Laboratory, The Francis Crick Institute, London, NW1 1AT, UK; c Department of Genetics, Moorfields Eye Hospital NHS Foundation Trust, London, EC1V 2PD, UK; d Department of Ophthalmology, Great Ormond Street Hospital for Children NHS Foundation Trust, London, WC1N 3JH, UK

**Keywords:** Usher syndrome, USH2A, Retinitis pigmentosa, Usherin, Photoreceptor, Hair cells, Therapy, Disease models

## Abstract

Bilallelic variants in the *USH2A* gene can cause Usher syndrome type 2 and non-syndromic retinitis pigmentosa. In both disorders, the retinal phenotype involves progressive rod photoreceptor loss resulting in nyctalopia and a constricted visual field, followed by subsequent cone degeneration, leading to the loss of central vision and severe visual impairment. The *USH2A* gene raises many challenges for researchers and clinicians due to a broad spectrum of mutations, a large gene size hampering gene therapy development and limited knowledge on its pathogenicity. Patients with Usher type 2 may benefit from hearing aids or cochlear implants to correct their hearing defects, but there are currently no approved treatments available for the *USH2A*-retinopathy. Several treatment strategies, including antisense oligonucleotides and translational readthrough inducing drugs, have shown therapeutic promise in preclinical studies. Further understanding of the pathogenesis and natural history of *USH2A*-related disorders is required to develop innovative treatments and design clinical trials based on reliable outcome measures. The present review will discuss the current knowledge about *USH2A*, the emerging therapeutics and existing challenges.

## Highlights

• Genetic variants and genotype-phenotype correlations of *USH2A*-retinopathy.
• Models and disease mechanisms of *USH2A*-retinopathy.
• The emergence of genetic therapies for syndromic and non-syndromic *USH2A*-retinopathy.

## Abbreviations

AAV: adeno-associated virus
AON: antisense oligonucleotide
dpf: days post-fertilization
ERG: electroretinography
hiPSC: human induced pluripotent stem cell
OCT: optical coherence tomography
NMD: nonsense-mediated mRNA decay
NsRP: non-syndromic retinitis pigmentosa
PMC: periciliary membrane complex
RdCVF: rod-derived cone viability factor
TRIDS: translational read-through inducing drugs

## 1 Introduction

*USH2A* mutations alone are the leading cause of autosomal recessive non-syndromic retinitis pigmentosa (nsRP), accounting for 12–25% of cases (McGee et al., 2010; Verbakel et al., 2018). RP has an estimated prevalence of 1 in 4000 people and is one of the most common causes of blindness amongst working age adults (Hartong et al., 2006; Pagon, 1988; Verbakel et al., 2018). This inherited retinal disease involves progressive photoreceptor loss; the rod photoreceptors are the first cells to degenerate, leading to night blindness and a constricted visual field. Eventually, the peripheral and foveal cone populations also decline, leading to the loss of central vision and severe visual impairment.

Simultaneously, *USH2A* mutations represent the most frequent cause of Usher syndrome, an autosomal recessive disorder involving dual impairment of the visual and audiovestibular systems. It was first described in 1858 by Albrecht Von Graefe (1858) and classified as an inherited disorder in 1914 by the Scottish ophthalmologist Charles Usher (1914). It is the most common cause of deaf-blindness with an estimated prevalence of 3–6 in 100,000 people worldwide (Toms et al., 2015). Patients with Usher syndrome have congenital sensorineural hearing loss with or without vestibular dysfunction, and visual loss in the form of RP. These symptoms allowed clinicians to classify the disease into three clinical subtypes depending on the severity of the hearing loss as well as on the presence of vestibular symptoms (Smith et al., 1994).

**Fig. 1:**
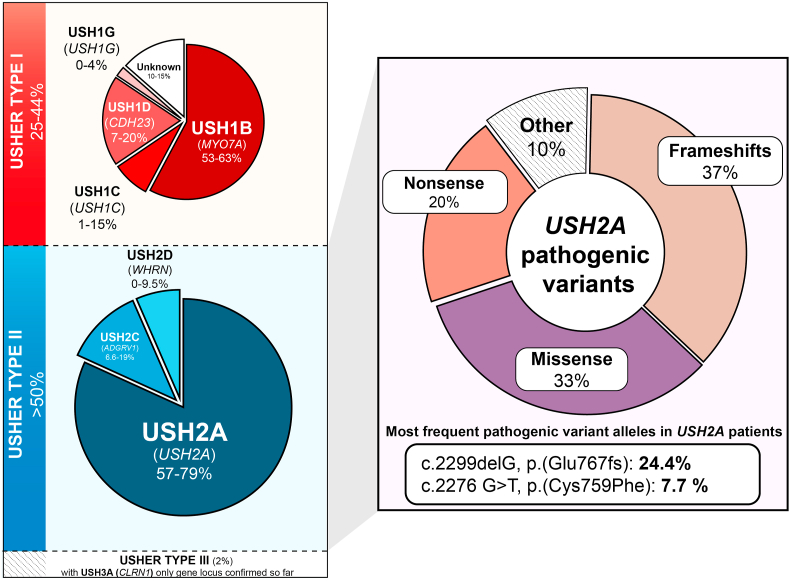
Diagram summarizing the prevalence of the Usher syndrome subtypes and *USH2A* pathogenic variants.

Usher syndrome type 1 is the most severe subtype characterized by severe-to-profound congenital deafness, vestibular dysfunction and prepubertal onset of RP in the first decade of life. So far, nine gene loci have been identified as involved in Usher type 1; USH1B (OMIM #276903) (*MYO7A*, myosin VIIa), USH1C (OMIM #605242) (*USH1C,* harmonin), USH1D (OMIM #605516) (*CDH23*, cadherin-23), USH1E (OMIM #602097) (*USH1E,* unknown), USH1F (OMIM #605514) (*PCDH15,* protocadherin-15), USH1G (OMIM #607696) (*USH1G,* SANS), USH1H (OMIM #612632) (*USH1H,* unknown), USH1K (OMIM #614990) (*USH1K*, unknown) (Toms et al., 2015) (Fig. 1). Recently, USH1M (OMIM #606351) (*ESPN,* Espin) has been reported in a family with an Usher type 1 phenotype (Ahmed et al., 2018). Mutations in *CIB2* (OMIM #605564), previously identified as causing USH1J, have subsequently been suspected to cause autosomal recessive non-syndromic hearing loss and not Usher syndrome (Booth et al., 2018).

Usher syndrome type 2 is the most common form accounting for more than 50% of all cases and exhibits a milder phenotype with moderate-to-severe hearing defects without vestibular dysfunction, and later onset of RP in the second decade of life. In total, three gene loci have been reported as associated with Usher type 2; USH2A (OMIM #608400) (*USH2A*, usherin) the most prevalent accounting for around 57–79% (Lentz and Keats, 1993) of this subtype, USH2C (OMIM #602851) (*ADGRV1*, ADGRV1 also known as VLGR1 or GPR98) and USH2D (OMIM #607928) (*WHRN*, Whirlin).

Lastly, Usher type 3 is the rarest form of Usher Syndrome, involving progressive hearing loss, variable vestibular dysfunction and onset of RP symptoms. Only one gene locus has been described for this subtype, USH3A (OMIM #606397) (*CLRN1*, Clarin-1).

## 2 Genetics of *USH2A*

The *USH2A* gene (OMIM #608400) is located on the long arm of chromosome 1q41, and codes for multiple usherin isoforms due to an alternative splicing, including a short isoform (a) containing 21 exons leading to a 1546-aa secreted protein (Eudy et al., 1998) and a very large isoform (b) with 51 additional exons (van Wijk et al., 2004). Isoform (b) is predominant in the retina and the cochlear, giving rise to a 5202-aa matrix protein with a predicted total molecular weight of 570 kDa. The long isoform b contains an intracellular region which interacts with the Usher protein network, a short transmembrane domain and a very long extracellular domain with several motifs associated with extracellular matrix proteins such as laminin and fibronectin repeats as represented in Fig. 3 (van Wijk et al, 2004, 2006). In addition to these two isoforms, a modified exon 71 encoding an additional 24-aa peptide restricted to the inner ear, has also been described (Adato et al., 2005).

The mutation spectrum is very heterogenous and includes over 1500 mutations with more than 690 variants presumed to be pathogenic (LOVD Database, accessed on September 10th, 2020) which span the whole *USH2A* gene, consisting of nonsense, missense, deletions, duplications, splicing variants and pseudo-exon inclusion variants (Baux et al., 2014). Most are private, but several are more prevalent such as the recurrent c.2299delG, pGlu767Serfs*21 (rs80338903) variant (Eudy et al., 1998; Liu et al., 1999; Weston et al., 2000). This is the most frequent pathogenic mutation (Le Quesne Stabej et al., 2012) and responsible for approximatively 24.5% (606/2484) pathogenic variants of *USH2A* on the LOVD database. The c.2299delG mutation has been reported in patients from Northern and Southern Europe, North America, South America, North and South Africa, and China (Baux et al., 2014) with a particular high allelic frequency of 30.6% in Scandinavia (Dreyer et al., 2008). Investigating the reasons underlying its exceptionally high prevalence through haplotype studies has found evidence of a European common ancestor (Aller et al., 2010; Dreyer et al., 2001). This single pair deletion in exon 13, which codes for the fifth laminin-type epidermal growth factor-like domain, generates a premature stop codon presumed to result in a truncated protein and/or nonsense-mediated mRNA decay (NMD). Additionally, it has been demonstrated to induce an exonic splicing defect leading to the skipping of exon 13 or both exons 12 and 13 (Lenassi et al., 2014).

Another common pathogenic variant, also located in exon 13, is the missense mutation c.2276 G > T, p. Cys759Phe (rs80338902) accounting for 7.6% of the pathogenic variants according to the LOVD database (191/2484). The replacement of a cysteine by a phenylalanine has been predicted to disrupt a presumed disulphide bond or lead to the rearrangement of a key region promoting interactions with the extracellular matrix, impairing the function of usherin (Pérez-Carro et al., 2018; Rivolta et al., 2002). A further relatively frequent mutation driving a diseased phenotype by a different mechanism is the deep-intronic c.7595-2144 A > G mutation (rs786200928) in intron 40 of *USH2A*. It induces the addition of a 152 bp pseudo-exon (PE) into the mature *USH2A* transcript between the exons 40 and 41 (Vaché et al., 2012). The *USH2A* transcript containing the aberrant exon is then subjected to the NMD pathway and further degradation (Slijkerman et al., 2016a). Additionally, the c.11864G > A, p. Trp3955* variant (rs111033364) is one of the most common variants according to the LOVD database (107/2637) and was the most frequent pathogenic variant in the Neuhaus et al. (2017) cohort.

In addition to *USH2A* variants, mutations in *PDZD7* gene (PDZ domain-containing 7) have been proposed to act as a retinal disease modifier in *USH2A* patients*,* explaining the frequently observed variability of the visual phenotype (Ebermann et al., 2010).

## 3 Understanding *USH2A* genotype-phenotype correlations

Mutations in *USH2A* are responsible for both Usher syndrome type 2 and nsRP, which are distinguished by impaired and preserved hearing function, respectively. Both disorders show autosomal recessive inheritance and therefore patients must carry two pathogenic allelic variants to drive a diseased phenotype. Accordingly, genotype-phenotype studies have been conducted to understand whether specific mutations were more likely to preserve hearing or to produce a more severe phenotype. Lenassi and colleagues have proposed a model of an allelic hierarchy, where the presence of at least one retinal disease specific *USH2A* allele results in the preservation of normal hearing and leads to nsRP. Retinal disease specific alleles were more likely to be those where some protein function may be preserved and thus, considered as phenotypically dominant to Usher syndrome type 2 alleles. For instance, carriers of at least one copy of c.2276 G > T, p. Cys759Phe (rs80338902) missense mutation are associated with nsRP with preserved hearing function (Lenassi et al., 2015b). Consistently, the truncating c.2299delG variant was showed to lead to a more severe and frequent hearing loss when compared to c.2276 G > T variant (Blanco-Kelly et al., 2015; Lenassi et al., 2015b).

In addition to these considerations, the onset and severity of the symptoms have also been investigated. The presence of two null alleles has been described as likely to cause more severe hearing loss and retinal degeneration (Pierrache et al., 2016). In a large *USH2A* patient cohort, nsRP patients were found to become visually impaired 13 years later based on visual field and 18 years later based on visual acuity than Usher syndrome type 2 patients. In addition, other studies have shown that the combination of two truncating mutations in *USH2A* causes more severe and progressive hearing impairment compared to the presence of one or two non-truncating mutations (Abadie et al., 2012; Hartel et al., 2016). Overall, it appears that remnant usherin protein function could attenuate both retinal and hearing symptoms. However, comparison of structural measurements (ellipsoid zone line length, horizontal diameter and vertical diameter) on retinal imaging between syndromic and non-syndromic *USH2A* patients showed no significant differences (Sengillo et al., 2017a). Consistently, a recent study investigating the patterns of degeneration between syndromic and non-syndromic RP patients using hyperautofluorescence ring, area horizontal diameter and ellipsoid zone width measurements, showed no significant differences between the two groups (Dubis AM et al. Invest Ophthalmol Vis Sci. 2019; 60:ARVO E-Abstract 5172).

Interestingly, differences in cone function have been identified when comparing electroretinogram (ERG) responses of syndromic and non-syndromic *USH2A*-related RP (Sengillo et al., 2017b). The Usher type 2 patients displayed a more attenuated 30 Hz-flicker amplitude of 17 μV compared to 2.1 μV in nsRP patients, indicating a reduced cone function in Usher 2 patients compared to those with nsRP. Nevertheless, an identical *USH2A* genotype may also lead to very different phenotypes between patients. A case report of two siblings carrying the same *USH2A* mutations displayed surprising clinical heterogeneity; one sibling was diagnosed with Usher syndrome 2 while the second sibling had completely preserved visual function, which was the first reported *USH2A-*related non-syndromic deafness (Lenassi et al., 2015a). This intra-familial genotype-phenotype discrepancy indicates the involvement of protective environmental, genetic or epigenetic factors.

## 4 Usherin protein – Localization, interacting partners and functions

**Fig. 2:**
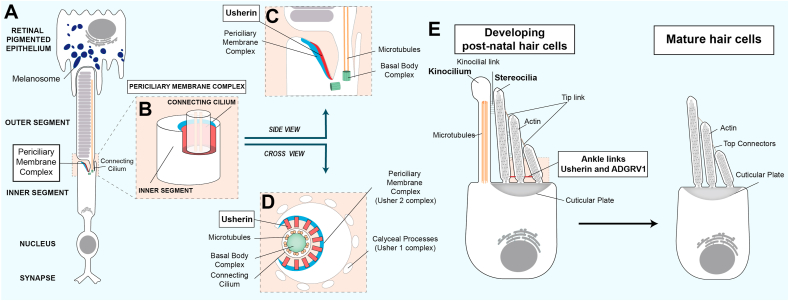
**Schematic diagram of usherin localisation in photoreceptors and hair cells.** (A) Cellular organisation of a photoreceptor. The photoreceptor possesses an inner segment and an outer segment, a highly specialised cilium responsible for light detection. The inner segment is connected to the outer segment through the connecting cilium. (B) The connecting cilium is wrapped in the periciliary membrane complex (blue), where the usherin long isoform (red) is spatially restricted. (C) Side view and section (D) of the periciliary membrane complex. (E) Hair cells are the ciliated sensory cells of the cochlea responsible for the transformation of sound-induced vibrations into electric signals. Usherin (red) is localised to the ankle link of developing post-natal hair cells. (For interpretation of the references to colour in this figure legend, the reader is referred to the web version of this article.)

The usherin long isoform b is predominantly expressed in the adult retina where it localises to the photoreceptor cells (Eudy et al., 1998; Huang et al., 2002; van Wijk et al., 2004). The photoreceptor, the light-sensitive cell of the retina, is composed of an inner segment and an outer segment. The inner segment is mainly responsible for the cell metabolism and protein production while the outer segment is a highly specialized cilia filled with stacks of discs containing photosensitive transmembrane proteins called opsins. The two structures are connected via the connecting cilium, a narrow collar wrapping around the photoreceptor ciliary axoneme (Fig. 2A). Here, Liu and colleagues were the first to show evidence of a specific usherin long isoform expression in murine photoreceptors, which was spatially restricted to the periciliary membrane complex (PMC), a structure wrapping the photoreceptor connecting cilium (Liu et al., 2007). In addition, usherin has been detected along the length of the connecting cilium of murine photoreceptor cells (Sorusch et al., 2017). Similarly, usherin has been specifically localized to the PMC of both cone and rod macaque photoreceptors (Sahly et al., 2012), zebrafish and Syrian hamster photoreceptors (Dona et al., 2018; Zou et al., 2019) (Fig. 2B–D).

**Fig. 3:**
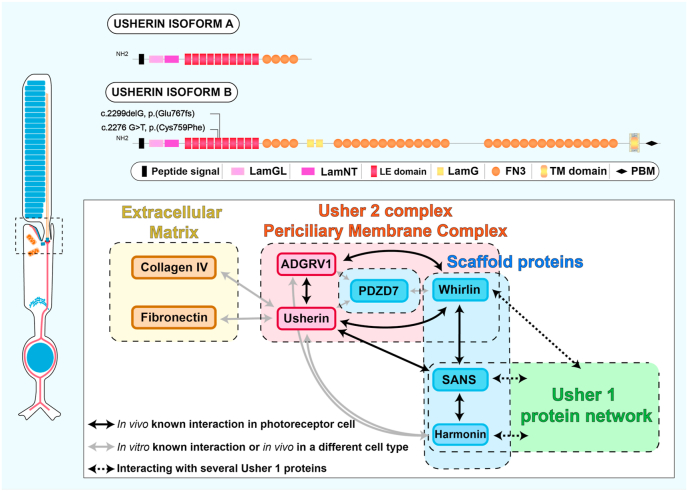
**Usherin isoforms and interacting partners in photoreceptors.** Usherin isoform *a* consists of 1 LamG-like jellyroll fold domain (LamGL), 1 Laminin N-terminal domain (LamNT), 10 laminin EGF-like domains (LE domain) and 4 fibronectin type III repeats. In addition to these domains, usherin isoform b is composed of 2 laminin G domains (LamG), 28 fibronectin type III repeats (FN3), a transmembrane domain (TM domain) and an intracellular PDZ-binding domain (PBM). The 2 most common mutations (c.2299delG, p.Glu767Serfs*21 and c2276 G>T, p.Cys759Phe) are located in the 5th laminin domain. Interacting partners have been divided into 4 groups: the Usher 2 complex periciliary membrane complex (red), the extracellular matrix partners (yellow), the scaffold proteins (blue) and the Usher 1 protein network (green). (For interpretation of the references to colour in this figure legend, the reader is referred to the web version of this article.)

The Usher interactome includes the Usher type 2 proteins, usherin, ADGRV1 (USH2C) and whirlin (USH2D) (Fig. 3). *In vitro* and *in vivo* experiments showed that whirlin physically interacts with both usherin isoform b and ADGRV1 isoform b through the association of the whirlin PDZ domain with usherin and ADGRV1 PDZ-binding domains (Reiners et al., 2006). This Usher 2 complex has been localized to the photoreceptor PMC in mice (Yang et al., 2010), zebrafish (Dona et al., 2018) and macaque models (Sahly et al., 2012).

Additionally, it was shown that removal of whirlin long isoform in a murine model resulted in the disruption of Usher 2 complex associated with usherin and ADGRV1 mislocalisation, and reduced usherin expression. Consistently, AAV-mediated whirlin replacement in this whirlin-knockout mouse model rescued both whirlin expression and Usher 2 complex localization to the PMC (Zou et al., 2011). In addition to its central role in Usher 2 complex formation, whirlin integrates the Usher 1 protein network by interacting with the unconventional actin-based motor protein myosin VIIa (USH1B), cadherin-23 (USH1D) and protocadherin-15 (USH1F), the scaffold protein SANS (USH1G) and the calcium/integrin binding-protein CIB2 (USH1J). Harmonin (USH1C), SANS (USH1G) and PDZD7, as well as whirlin, are key assembler scaffold proteins at the centre of the Usher protein network (Chen et al., 2014; Reiners et al., 2005a; Slijkerman et al., 2017; Sorusch et al., 2017; van Wijk et al., 2006; Yang et al., 2010; Zou et al, 2011, 2015). Harmonin has been shown to interact with all Usher 1 proteins (except CIB2) and is linked to the Usher 2 complex via ADGRV1 and usherin binding (Reiners et al., 2005b). Similarly, SANS interacts with the same Usher 1 proteins but also binds to both whirlin and harmonin. Moreover, SANS has been recently described as being part of a protein complex in the photoreceptors involving usherin and whirlin (Sorusch et al., 2017). Sorusch and colleagues hypothesized that SANS is involved in two distinct functions in photoreceptors; firstly, scaffolding the Usher complex for intracellular transport through the inner segment and secondly, regulating the cargo transfer from the transport machinery of inner segment to the ciliary transport module at the base of the photoreceptor cilium (Sorusch et al., 2017).

It remains unknown why the rod photoreceptors degenerate before cones in the *USH2A*-retinopathy, despite usherin being expressed in both cell types. Recently, single-cell RNA sequencing from adult human retinas revealed *USH2A* expression was photoreceptor-specific, but at higher levels in the cones compared to the rods (Cowan et al., 2019). It is possible that usherin function in cone photoreceptors is less essential to their long-term maintenance compared to rods, or usherin function can be compensated for in cones; the discrepancy between the two cell types may help understand the pathogenesis behind *USH2A*-related RP. Further characterization of usherin protein interacting partners, their function and subcellular localization will aid in understanding the role of usherin and *USH2A*-related pathogenesis mechanisms.

In light of *USH2A*-related hearing symptoms, the subcellular distribution of the usherin isoform b has been characterized in the ciliated sensory cells of the cochlea, known as hair cells (Fig. 2E). Located within the inner ear, sensory hair cells accommodate a mechano-transduction system converting sound-induced vibrations in electric signals (Schwander et al., 2010). The hair bundle, a set of highly specialized microvilli (known as stereocilia) protruding from the hair cell apical surface, is responsible for hair cell mechano-sensitivity. Vibration-induced deflection of these stereocilia leads to the opening of a mechano-sensitive ion channel, resulting in depolarization of the hair cell. Usherin isoform b has been localized to the ankle links of the developing cochlear hair cells (Adato et al., 2005; Liu et al., 2007; Michalski et al., 2007). The ankle links form transient fibrous structures spanning between the bases of growing stereocilia, essential for correct development and organization of the hair bundle. In the developing mouse hair cells, their development occurs at postnatal day 0–2 (P0–P2) and persists until P9. By P12, the ankle links are no longer detectable (Goodyear et al., 2005). Consistently, several studies have reported similar transient expression patterns of usherin long isoform and ankle links (Adato et al., 2005). Furthermore, the three Usher 2 proteins are interdependent for preservation of normal subcellular localization at the ankle links (Adato et al., 2005). The scaffolding protein PDZD7 has also been localized to the stereociliary ankle links of developing hair cells (Grati et al., 2012) coupled with *in vitro* affinity properties for the Usher 2 complex (Chen et al., 2014). Hence, PDZD7 appears as a potential component of the Usher 2 complex, especially when considered with the finding that a frame-shift mutation in *PDZD7* combined with homozygous *USH2A* mutations was associated with a more severe *USH2A*-related phenotype (Ebermann et al., 2010).

Hair cells and photoreceptor cells involved in *USH2A*-related disorders are sensory-ciliated cells. In hair cells, the stereocilia on their apical surface are microvilli-structures while the genuine axonemal cilium is the kinocilium, which is resorbed during hair cell development (Leibovici et al., 2005; Sobkowicz et al., 1995). It plays a critical role in hair cell V-shaped stereociliary bundle development and orientation (Jones and Chen, 2008; Schwander et al., 2010). Similarly, two comparable structures are found in photoreceptors, where the true cilium is the outer segment while the microvilli are the calyceal processes forming a collar around the lower half of the outer segment (O'Connor and Burnside, 1981; Sahly et al., 2012). Interestingly, the calyceal processes are conserved in frog, zebrafish and primate photoreceptors. The Usher 1 complex has been localized to the photoreceptor calyceal processes, suggesting a role in retinal structure maintenance (Sahly et al., 2012). Similar to its role in ankle links for the correct development of hair bundles, usherin could span between the apical inner segment and the connecting cilium internal membrane to strengthen or arrange a proper spacing, ensuring correct maintenance of connecting cilium and outer segment. (Maerker et al., 2008; Sorusch et al., 2017). Additionally, associations with extracellular matrix components could be involved in its structural maintenance function. Supporting this idea, interactions of usherin LE domain with type 10.13039/501100000026IV collagen 7 S domain and fibronectin have been characterized (Bhattacharya et al., 2004; Bhattacharya and Cosgrove, 2005).

In addition to retinal and cochlear expression, it has been shown that usherin isoforms are expressed in other tissues. The short isoform is secreted in the extracellular matrix and has been described in testis, small and large intestines, uterus and ovary, while the long isoform has been reported in heart and kidneys (Bhattacharya et al., 2002; Pearsall et al., 2002; van Wijk et al., 2004). As Usher syndrome has been proposed to act as a ciliopathy, investigations towards other ciliated cells has been conducted (Sorusch et al., 2014; Tsang et al., 2018; Wolfrum and Nagel-Wolfrum, 2018). For instance, sperm abnormalities have been observed in patients with Usher type 2 associated with atypical axoneme leading to decreased sperm motility and velocity. Although contradictory studies have been subsequently reported (van Aarem et al., 1999), one study indicated cilia axoneme impairment in sperm as part of the Usher syndrome pathogenesis (Hunter et al., 1986). Further evidence implicating Usher syndrome as a ciliopathy is the accelerated age-related olfactory decline reported among Usher patients (Ramos et al., 2019; Ribeiro et al., 2016). Additionally, a recent study showed reduced ciliogenesis in *USH2A* patient-derived human dermal fibroblasts compared to a healthy control (Samanta et al., 2019).

## 5 *USH2A* models

The accumulated knowledge regarding the genetic and clinical characterization of *USH2A-*related disease has allowed the generation of cellular and animal models. Such models have provided invaluable insights into the *USH2A*-related disease pathogenesis and are essential tools to assess new therapeutic strategies.

### 5.1 Cellular models

To circumvent the limited supply of *USH2A* patient retinal primary cells, human induced pluripotent stem cell (hiPSC) technology offers an unlimited source of retinal tissue preserving a human and patient genetic background. hiPSCs are pluripotent stem cells generated from patient-derived somatic cells, which have the potential to differentiate into any cell type of the body, including retinal cells (Takahashi and Yamanaka, 2006). To achieve this, several *in vitro* differentiation protocols of hiPSCs towards a retinal fate have been optimized, leading to the generation of 3D self-organizing optic cup-like structures, known as retinal organoids (Llonch et al., 2018). These *in vitro* models can give rise to organized laminated layers containing all major retinal cell types. Furthermore, the retinal organoids not only follow the naturally occurring *in vivo* retinal development in a stepwise fashion (Meyer et al., 2009) but also display the same transcriptional signature as a normal developing human retina (Cowan et al., 2019; Welby et al., 2017). Significantly, the hiPSC-derived cone and rod photoreceptors contain rudimentary outer segment-like structures, capable of light-responses (Hallam et al., 2018; Zhong et al., 2014). Overall, these characteristics suggest that retinal organoids are relevant models for the human retina.

The derivation of hiPSCs from patients with *USH2A*-related disease offers the opportunity to create the ‘disease in a dish’ and thus dissect the underlying molecular mechanisms and screen innovative therapies. To date, only two publications have reported the generation of *USH2A*-retinal cells derived from hiPSCs*.* The first study conducted by Tucker et al. (2013) used a 3D/2D protocol to produce eye cup-like structures derived from keratinocytes cells from a patient carrying the deep-intronic c.7595-2144 A > G mutation (rs786200928) in intron 40 of *USH2A* and the c.12575G > A mutation (rs199605265) *in trans*. While there were no obvious differences indicative of early developmental abnormalities in *USH2A*-derived retinal cells compared to the control, they displayed an increased GRP78 and GRP94 protein expression levels, suggesting that ER stress could be involved in *USH2A*-related pathogenesis (Tucker et al., 2013).

The second study by Guo et al. (2019) produced retinal organoids derived from reprogrammed urine cells of a patient with nsRP carrying the c.9127-9129delTCC and c.8559-2 A > G (rs397518039) mutations in *USH2A*. In contrast to the previous study, the investigators found reduced laminin expression, defective retinal progenitor cell differentiation and disorganized neural retina, with higher expression of pro-apoptotic genes and decreased expression of cilia-associated genes in patient-derived retinal organoids compared to wild-type controls (Guo et al., 2019). However, these findings were produced from 12-week-old retinal organoids, and usherin expression has not been demonstrated at this time point. In addition, no evidence of interaction between the usherin and its partners such as whirlin or ADGRV1 was provided. More mature retinal organoids would have been more relevant, as supported by single-cell transcriptomic data analysis of retinal organoids exhibiting a high *USH2A* gene expression in 24-week -old retinal organoids (Cowan et al., 2019).

Although limited *USH2A*-related patient-derived retinal organoids have been published, several retinal organoids modelling ciliopathy-related retinal diseases have successfully recapitulated key features of patient retinal phenotype, such as *CEP2*90-LCA related ciliopathy (Parfitt et al., 2016), *RP2*-related *RP* (Schwarz et al., 2017) and *RPGR*-associated *RP* (Deng et al., 2018).

Although they have great potential for advancing our knowledge around *USH2A*-related retinal disease, many challenges remain to be addressed to improve retinal organoid models. Firstly, the generated photoreceptors are not fully developed, with limited outer segment formation and disc organization, which hampers the understanding of molecular mechanisms and architectural maintenance of photoreceptor cells. In addition, hiPSCs display a high variability to produce layered retinal organoids with photoreceptor cells, and can be affected by the patient, the hiPSC clone and the retinal differentiation protocol (Cowan et al., 2019; Mellough et al., 2019).

### 5.2 Animal models

Mouse models are a highly valuable tool in ophthalmology research, as demonstrated by the numerous retinal degenerative mice models that have been characterized (Veleri et al., 2015). As mammals, mice share a high level of gene conservation with humans, with a similar retinal organization and function. However, owing to their nocturnal lifestyle, the murine retina contains far fewer cone photoreceptors than the human retina, and they also do not possess any enriched cone regions, such as the fovea, found in primate retina (Slijkerman et al., 2015).

An *Ush2a*-null mouse model has been described, displaying complete depletion of both usherin isoforms in the retina and the cochlear (Liu et al., 2007). *Ush2a*-null mice exhibited a late-onset photoreceptor degeneration and a non-progressive moderate hearing loss at high frequencies, without vestibular dysfunction, recapitulating the main symptoms found in the patients with Usher type 2. Loss of outer hair cells bundles and mislocalisation of usherin partners such as whirlin, PDZD7 and ADGRV1 were observed in the mutant cochlea (Zou et al., 2015). Up to 10 months of age, no phenotypic differences in retinal structure and function were observed in *Ush2a*-null mice compared to wild-type, although Müller cell activation in the mutant retina was described as early as 2 months post-natally, indicative of early retinal stress (Liu et al., 2007). By 20 months of age, the photoreceptor nuclei were reduced by half and accordingly, ERG responses were significantly reduced compared to the age-matched wild-type mice, demonstrating a late-onset progressive retinal degeneration (Liu et al., 2007). Interestingly, further studies showed red-green cone opsin mislocalisation at P80 in the *Ush2a* mutant mice, prior to widespread degeneration (Lu et al., 2010); however, the mechanisms underlying the retinal changes have not been elucidated in mice.

Another Usher-like mouse model named KM^USH/USH^, displaying spontaneous RP and moderate hearing loss, showed decreased expression in both *Pde6b* and *Ush2a* gene (Yao et al., 2016). Several point mutations were identified in the *Ush2a* gene, suggesting a causative role in the KM^USH/USH^ usher-like phenotype. However, further studies showed that a single base deletion occurring in *Adgrv1* was responsible for the hearing loss phenotype of this model (Yan et al., 2018).

Although retinal degeneration has been reproduced in *Ush2a*-null mice, a high phenotypic discrepancy has been described when comparing human patients and other Usher mutant mice. Accordingly, only *Ush2a-*null and *Whirlin*-null mutant mice displayed obvious retinal degeneration (Toms et al., 2015). One of the main reasons for the discrepancy between Usher patients and mice could be the absence of calyceal processes in murine photoreceptors, where the Usher 1 proteins have been found to localize in other species. The calyceal processes have been proposed to form an adhesion belt from the apical inner segment to the outer segment basal region in primate retinas (Sahly et al., 2012). Furthermore, findings in *Xenopus* have indicated that calyceal processes, together with their associated links, may control the sizing of rod discs and cone lamellae throughout their daily renewal (Schietroma et al., 2017).

Zebrafish (*Danio rerio*) are alternative vertebrate models becoming increasingly popular for research into inherited ocular diseases (Angueyra and Kindt, 2018). Human and zebrafish eyes share several common features; the structural organization of the retina is well-conserved with a cone-enriched retina (Richardson et al., 2017) responsible for acuity and colour vision. In addition, 70% of human genes have at least one zebrafish orthologue, allowing the recapitulation of disease phenotypes in mutant models (Howe et al., 2013). Regarding the *USH2A* gene, the zebrafish and human usherin protein sequences show 52% identity and 68% similarity with the same domain structure (Dona et al., 2018), supporting a conserved role of usherin between these species.

To date, five different *ush2a* zebrafish models have been generated. Dona and colleagues characterized two *ush2*a zebrafish lines (*ush2a*^*rmc1*^ and *ush2a*^*b1245*^) with protein-truncating mutations, generated using CRISPR/Cas9 (Dona et al., 2018). The mutant zebrafish retinas showed a complete ablation of usherin expression along with a reduction of the other Usher 2 proteins, adgrv1, whirlin A and B, at the photoreceptor PMC. In addition, ERG responses were reduced at 5 days post-fertilization (dpf) compared to age-matched wild-type zebrafish. The photoreceptor degeneration in the larval retina was exacerbated by constant high-level light exposure of 3000 lux up to 8 dpf. Similarly, Han and colleagues reported a further *ush2a*-null zebrafish (*ush2a*^*hzu6*^) generated using TALEN technology, displaying early retinal dysfunction (at 6–7 days post-fertilization) demonstrated by ERG recordings (Han et al., 2018). Furthermore, levels of rod-specific proteins rhodopsin were reduced from 7 months and shortening of photoreceptor outer segments was described from 12 months. Surprisingly, in contrast to all previous studies, usherin expression in wild-type zebrafish was detected in the retinal ganglion cell layer as well as the photoreceptors. Additionally, *ush2a*^*hzu6*^ larvae showed decreased acoustic startle responses indicative of early impaired auditory function.

Recently, the novel CRISPR/Cas9-generated *ush2a*^*u507*^ zebrafish mutant line was described; unlike the previously reported mutants (*ush2a*^*rmc1*^, *ush2a*^*b1245*^ and *ush2a*^*hzu6*^) displaying early defects, this line presented a slowly progressive adult retinal degeneration, with increased apoptotic photoreceptor levels from 6 months post-fertilization (Toms et al., 2020). Rhodopsin and blue opsin mislocalisation with lysosome-like structures in the photoreceptors were also observed from 6 months post-fertilization. Following these results, further characterization of the *ush2a*^*rmc1*^ revealed a similar pattern of photopigment mislocalisation with elevated autophagy levels at 6 dpf. Defective photopigment trafficking is consistent with the hypothesis of the Usher 2 complex playing a role in docking and fusion of transport vesicles through the connecting cilium to the outer segment (Toms et al., 2020).

In addition the four *ush2a*-knockout models described, a humanized zebrafish knock-in model for the deep-intronic c.7595-2144 A > G mutation in *USH2A* has been generated (Slijkerman et al., 2018). Even though only 7.4% of *ush2a* transcripts contained the human pseudo-intron and did not reveal phenotypic changes compared to wild-type zebrafish, antisense-morpholino treatment was able to partially correct the *ush2a* aberrant splicing. Despite some limitations in zebrafish use, including the duplication and functional redundancy of some genes and species differences in ocular anatomy, these models are invaluable tools to better understand *USH2A*-related pathogenesis and to provide preclinical proof-of-concept for the development of efficient and safe treatments.

## 6 Therapeutic strategies

**Fig. 4:**
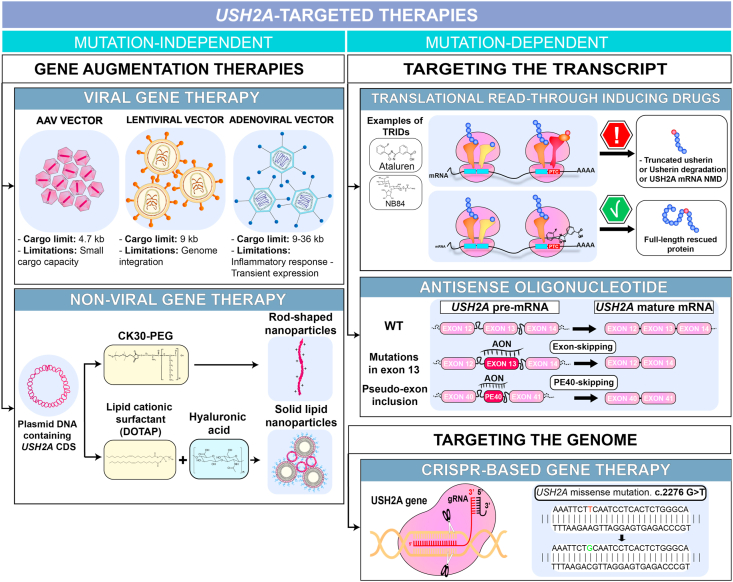
**Therapeutic strategies for *USH2A*-related disease.***USH2A*-targeted strategies can be divided into two categories, mutation-independent and -dependent. The mutation-independent can be applied to all the *USH2A*-related diseases regardless of the pathogenic mutation. It involves the generation of healthy copies of *USH2A* coding sequence (CDS) using viral or non-viral vectors. AAV and lentiviral vectors cannot accommodate the *USH2A* CDS while adenoviral vectors can. Mutation-dependent strategies have to be applied to a specific mutation or type of mutation. It can target the transcript using translational read-through inducing drugs (e.g. nonsense mutation) and antisense oligonucleotides (e.g. pseudo-exon inclusion), or target the genome using CRISPR-Cas9 editing (e.g. missense mutation). AAV, adeno-associated vector; CK30-PEG, 30-mer polylysine conjugated with polyethylene glycol; DOTAP, 1,2-dioleoyl-3-trimethylammonium-propane; TRIDs, translational read-through inducing drugs; NMD, nonsense-mediated decay.

To date, no approved treatments are available to alleviate the retinal symptoms of *USH2A-*related disease. Potential therapeutic strategies can be divided in two main types: *USH2A*-targeted therapy, which manipulate levels of *USH2A*; and *USH2A*-independent therapy, which aims to prevent retinal degeneration through targeting common disease pathways such as cell death or oxidative stress (Fig. 4).

Gene therapy

It has been almost 50 years since the concept of introducing genetic material into a host cell for therapeutic purposes was formulated by researchers. Gene therapy has had to overcome many pitfalls to reach the clinic, and still has many challenges to address. Regarding the treatment of inherited eye diseases, the eye benefits from its immune-privileged status, accessibility and compartmentalization allowing a restricted spread of the delivered drug.

**Fig. 5:**
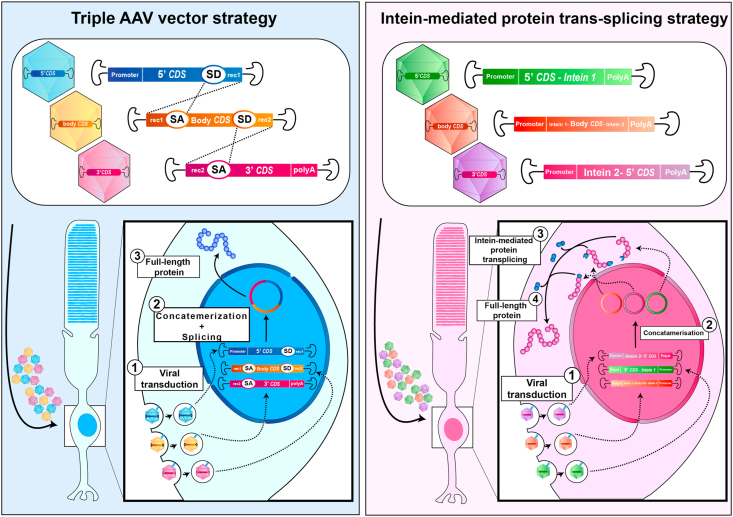
**Expanding AAV vector capacity for large gene transfer.** The scheme represents two strategies that allow the successful transfer of large genes. The first strategy on the left panel is based on the concatemerisation and splicing of three transgenes to reconstitute the full-length coding sequence of interest. AAV vectors carrying the three different transgenes transduce the photoreceptor cell. The transgenes concatemerise and splice into a single episome in the nucleus allowing the production of the full-length protein of interest. The second strategy consists of intein-mediated transplicing allowing the reconstitution of the full-length protein. Similarly to the previous strategy, AAV vectors carrying three different transgenes transduce the targeted cell. However, it forms three distinct episomes. The three proteins resulting from these episomes by intein-mediated transplicing, lead to the full-length protein. AAV, adeno-associated virus; CDS, coding sequence; SD, splicing donor; SA, splicing acceptor; rec, recombinogenic region.

Viral gene therapy using adeno-associated viruses (AAVs) has proven to be the safest and most effective option for transducing cells in a retinal context so far (Ramlogan-Steel et al., 2019). Indeed, Voretigene neparvovec gene therapy developed by Spark Therapeutics was recently approved by both the European Medicine Agency (EMA) and the U.S Food and Drug Administration (FDA); this AAV vector-based therapy was developed for the treatment of Leber congenital amaurosis patients with confirmed biallelic *RPE65* mutations (Russell et al., 2017). Although the transduction efficacies of several engineered AAVs with enhanced capsids towards photoreceptor cells have been well documented, the AAV cargo limit is still 4.7 kb. Single AAV particles cannot carry the full *USH2A* coding sequence (15.6 kb). Therefore, several strategies have been investigated and optimized to overcome AAV carriage limitations. The use of dual and triple AAVs has expanded the transfer capacity from 4.7 kb to a maximal capacity of 14 kb (Maddalena et al., 2018; Trapani et al., 2014). However, this technology displays a limited efficiency, reaching 40% efficacy of a single AAV photoreceptor transduction in pig retina, and still does not provide enough capacity for *USH2A* cDNA. Recently, Maddalena and colleagues used intein-mediated protein trans-splicing to expand AAV transfer capacity in the retina. Inteins are genetic elements leading to the production of splice sites in proteins (Maddalena et al., 2018). They allow the protein to excise itself to produce a full-length protein without leaving any amino acid modifications in the final product or without an external energy source. This technology allowed them to efficiently restore ABCA4 (6.8 kb-long transcript) and CEP290 (7.4 kb-long transcript) protein levels in the retina of two corresponding mouse models. A higher efficiency of transduction was achieved compared to the triple AAV strategy (Maddalena et al., 2018). Nevertheless, intein-mediated protein trans-splicing via AAV vectors requires the use of cis-regulatory sequences in each AAV, limiting its actual capacity. To translate this strategy to *USH2A*, more than 5 AAV particles transducing the same photoreceptor cell to produce the full usherin protein would be needed (Fig. 5).

To accommodate the full-length *USH2A* coding sequence, a viral vector with a large carrying capacity would be required. Lentiviral vectors have shown utility for gene therapy, resulting in the clinical trial evaluating the use of recombinant EIAV-based lentiviral vector *UshStat* for treating USH1B (*MYO7A*) patients (NCT01505062, NCT02065011). However, they have a gene size limit of 9 kb and therefore are not able to accommodate the full-length *USH2A* coding sequence (Lee et al., 2017). Alternatively, adenoviral vectors display a sufficient cargo capacity (8–36 kb) to carry the full *USH2A* cDNA, and so would be an ideal candidate for gene replacement. Furthermore, they can efficiently transduce mouse and human retinal cells, such as the Helper-dependent Adenovirus 5 (Han et al., 2019; Puppo et al., 2014). However, adenoviral vectors are prone to elicit a harmful inflammatory response leading to retinal damage. Developing strategies to circumvent innate immune responses would improve the translational development of such viral vectors for large transgene expression in ocular gene therapy.

Another approach for large gene sizes has been applied for Duchenne muscular dystrophy caused by the dystrophin gene (*DMD*) mutations (11 kb-long coding sequence where shorter synthetic dystrophin versions, known as microdystrophins, have been developed, allowing transduction by AAV vectors (Duan, 2018; Le Guiner et al., 2017). This strategy requires an extensive knowledge of the crucial protein domains to ensure a functional protein is produced. However, examining the broad mutation spectrum of *USH2A* has not highlighted domains that could be removed to decrease its size.

The limitations of viral vectors for gene therapy has encouraged efforts towards the development of alternative strategies. Non-viral synthetic vectors are easier to produce, less immunogenic and most importantly, suitable for larger transgenes. For instance, nanoparticles can accommodate DNA plasmid vectors up to 20 kb and do not limit effective *in vivo* gene transfer (Fink et al., 2006), which would accommodate the *USH2A* coding sequence. The use of compacted DNA nanoparticles formulated with polyethylene glycol-substituted polylysine (CK30PEG) containing the *ABCA4* cDNA cassette (6.8 kb) was able to improve the phenotype of an *abca4*-deficient mouse model, when delivered subretinally (Han et al., 2012). Additionally, the carrying capacity of nanoparticles allows the addition of cis-regulatory elements such as promoters, insulators and scaffold-matrix attachment region (S/MAR) sequences (Planul and Dalkara, 2017). However, despite extensive research, non-viral methods remain less efficient at transducing cells compared to viral vectors.

### 6.1 Gene-editing with CRISPR

As an alternative to gene augmentation therapy, the CRISPR/Cas9 breakthrough has paved the way for the use of gene-editing strategies for inherited diseases through correction of point mutations and small indels. It provides a promising option for treating diseases caused by large genes such as *USH2A* whether by correcting the *USH2A* mutation directly in the patient retina or by correcting hiPSC-derived photoreceptors for future transplantation.

To date, CRISPR/Cas9 targeting of *USH2A* involved *in vitro* editing of the recurrent c.2299delG, p. Glu767Serfs*21 mutation (rs80338903) and c.2276 G > T, p. Cys759Phe (rs80338902) in HEK cell models, patient-derived fibroblasts and hiPSC (Fuster-García et al., 2017; Sanjurjo-Soriano et al., 2019). Despite proof-of-concept *in vitro*, for clinical translation there remains many challenges such as eliminating off-targets, and increasing *in vivo* editing efficiency in post-mitotic cells such as photoreceptors (Burnight et al., 2018).

### 6.2 Translational read-through drugs (TRIDs)

As 20% of *USH2A* pathogenic variants are nonsense mutations, *USH2A*-related diseases are an ideal target for small translational read-through molecules (TRIDs). The introduction of a premature termination codon leads to either degradation of the mRNA by NMD or premature termination of the translation leading to a non-functional protein. Small compounds, such as ataluren (PTC124) or designer aminoglycosides (NB84), induce ribosomal translation infidelity, allowing a near cognate amino acid to compete with a release factor (Nagel-Wolfrum et al., 2016). This allows the premature termination codon to be bypassed, resulting in restored translation with synthesis of up to 25% of full-length protein levels. This approach has been investigated in Usher type 1 models (Rebibo-Sabbah et al., 2007; Welch et al., 2007). Notably for *USH1*C (harmonin) nonsense mutations, the efficacy of designer TRIDS, NB30, NB54 and PTC124, was investigated *in vitro* in HEK cells and in *ex vivo* mice retinal cultures allowing partial restoration of full-length functional protein (Goldmann et al, 2011, 2012).

For *USH2A*-related disease, Neuhaus and colleagues applied PTC124 to a HEK cell model containing a cDNA fragment from c.12,550–15996 of usherin long isoform containing the p. Trp3955* mutation and showed a 3.3 fold increase in *USH2A* expression compared to a DMSO control (Neuhaus et al., 2017). Further studies demonstrated PTC124 efficacy in restoring usherin expression and primary ciliogenesis capability in *USH2A* patient-derived fibroblasts with the p. Gly3142* (rs397518048) mutation (Samanta et al., 2019). Additionally, the EMAand FDA has granted Ataluren orphan drug designation for the treatment. Therefore, TRIDs show promise as a safe and cost-effective strategy to treat a range of *USH2A* nonsense mutations.

### 6.3 Antisense oligonucleotides

Antisense oligonucleotides (AON) are small and versatile RNA molecules that can interfere with mRNA splicing by specifically blocking aberrant splice sites and therefore allowing restoration of correct splicing (Crooke, 2017). Investigations into using AONs for the treatment of inherited retinal diseases have yielded encouraging results. A clinical trial is currently underway in which QR-110, an RNA antisense oligonucleotide for intravitreal injection, is being tested in patients with Leber congenital amaurosis carrying the deep-intronic mutation c.2991 + 1655 A > G in the ciliopathy gene centrosomal protein 290 (*CEP290*) (NCT03140969). The c.2991 + 1655 A > G mutation causes a splicing defect leading to a premature stop codon. QR-110 treatment showed restoration of the correct splicing and resulted in vision improvement at 3 months (Cideciyan et al., 2019).

Such strategies are applicable for *USH2A* patients carrying the deep-intronic c.7595-2144 A > G mutation (rs786200928) in intron 40 of *USH2A*, which introduces a pseudo-exon PE40 (Vaché et al., 2012). Slijkerman and colleagues demonstrated that administration of engineered AON to *USH2A* patient fibroblasts carrying the c.7595-2144 A > G mutation partially restores correct splicing of *USH2A* mRNA (Slijkerman et al., 2016b). In addition to the correction of deep-intronic mutations, an AON candidate (QR-421a) has been designed to exclude the whole exon 13 of the *USH2A* mature transcript (https://www.proqr.com/qr-421a-for-usher-syndrome-type-2/). Given that two of the most common pathogenic *USH2A* mutations are in exon 13 (LOVD Database), it has been hypothesized that QR421a treatment will result in restoration of a slightly shorter but functional usherin protein. A clinical trial is currently ongoing in patients with RP due to mutations in *USH2A* exon 13 (NCT03780257).

### 6.4 *USH2A*-Independent approaches

RP ultimately lead to irreversible degeneration of photoreceptors, with initial loss of rods later followed by cones. Gene independent strategies are being considered, which operate by either slowing down the degenerative process or by restoring retinal photosensitivity.

Neuroprotective strategies have been investigated to prevent photoreceptors degeneration in several models (reviewed in Pardue and Allen, 2018). For instance, the rod-derived cone viability factor (RdVCF), has been shown to slow the rate of cone cell death and to improve cone function in rat and murine models of retinal disease (Byrne et al., 2015). RdCVF, a truncated thioredoxin-like protein encoded by the nucleoredoxin-like-1 gene (*NXNL1*), is endogenously secreted by rod photoreceptors and promotes retinal cone survival by facilitating glucose uptake and metabolism. Therefore, loss of rods occurring in the first stage of RP results in cone death. By restoring RdCVF secretion, the cone photoreceptors responsible for the visual acuity of patients would be preserved.

Similarly, ciliary neurotrophic factor (CNTF) has been found to prolong photoreceptor survival in mouse and rat models of retinal degeneration (Li et al., 2010; LaVail et al., 1998, Liang et al., 2001, Ramlogan‐Steel et al., 2019). Currently, retinal implantation of capsules containing human NTC-201 cells releasing CNTF is in a phase 2 clinical trial for patients with RP (NCT00447980) (MacDonald et al., 2007; Talcott et al., 2011).

Recent investigations into neuroprotective strategies suggest that histone deacetylase (HDAC) inhibition slows rod and cone photoreceptor degeneration in different murine models of retinal dystrophies, revealing a promising alternative solution for cone-rod dystrophies (Mitton et al., 2014; Samardzija et al., 2019; Trifunović et al., 2016). However, the neuroprotective strategies for *USH2A*-related patients are only relevant in the early stages of RP. Once the photoreceptor layer is fully degenerated, strategies to replace photoreceptor function are required.

Among the gene-independent approaches, optogenetic therapies involve the introduction of light-sensitive proteins, named optogenes, into the remaining cells of the degenerative retina. It has already been shown that targeting the remaining cone cell bodies (Busskamp et al., 2010) with hyperpolarizing optogenes, bipolar cells or retinal ganglion cells (Berry et al., 2019; Macé et al., 2015; Sengupta et al., 2016) with depolarizing optogenes or middle-wave opsin, restores visual responses in mouse models of RP and post-mortem retina of macaque. However, targeting the remaining cone cell bodies with optogenes does not prevent the cell degenerating. Therefore, a combining this approach with a neuroprotective strategy such as RdCVF may be of benefit. Currently, two clinical trials are underway using AAV vectors to deliver the optogenes to the retinal cells of interest (NCT02556736 for advanced RP, and NCT03326336 for nsRP).

Finally, the accessibility of the eye makes it an ideal candidate for cell therapy and retinal prostheses for patients with advanced retinal degeneration. Embryonic stem cell and iPSC technology, as well as the extensive research that has led to the optimisation of retinal differentiation protocols, has improved the availability of tissue for transplantation. Photoreceptor transplantation aims to rebuild the photoreceptor layer by grafting *in vitro* generated photoreceptors (Gagliardi et al., 2019) but many challenges, such as the cell product manufacture process and the maintenance of the light-sensitive properties of the transplant, must be addressed. Innovative strategies such as the transplantation of optogenetically engineered photoreceptors have demonstrated promising results to ensure reliable light-sensitive properties of the graft (Garita-Hernandez et al., 2019).

## 7 Conclusion

More than 20 years after the discovery of the *USH2A* gene (Eudy et al., 1998) and 15 years after the usherin long isoform characterization (van Wijk et al., 2004), significant progress has been made in understanding *USH2A-*related disorders. Technical advances such as next-generation whole genome sequencing and non-invasive imaging has facilitated clinicians and geneticists in making *USH2A-*related diagnoses and the investigating of genotype-phenotype correlations. At the molecular level, even though its pathogenesis has not yet been elucidated, the recent emergence of *USH2A* cell and animal models generated using hiPSCs or CRISPR/Cas9 will aid in further unravelling the *USH2A* pathogenesis. Currently, several clinical trials are underway encompassing diverse strategies that could benefit *USH2A-*related patients.

## Funding

This work was funded by Santen Pharmaceutical Co., Retina UK and Wellcome Trust.

## Declaration of competing interest

The authors declare no competing interests.
